# Induction of the *CLOCK* Gene by E2-ERα Signaling Promotes the Proliferation of Breast Cancer Cells

**DOI:** 10.1371/journal.pone.0095878

**Published:** 2014-05-02

**Authors:** Liyun Xiao, Alan K. Chang, Ming-Xi Zang, Hailian Bi, Shujing Li, Miao Wang, Xinrong Xing, Huijian Wu

**Affiliations:** 1 School of Life Science and Biotechnology, Dalian University of Technology, Dalian, China; 2 School of Life Science and Medicine, Dalian University of Technology, Panjin, China; 3 Department of Biochemistry and Molecular Biology, School of Basic Medical Sciences, Zhengzhou University, Zhengzhou, China; McGill University, Canada

## Abstract

Growing genetic and epidemiological evidence suggests a direct connection between the disruption of circadian rhythm and breast cancer. Moreover, the expression of several molecular components constituting the circadian clock machinery has been found to be modulated by estrogen-estrogen receptor α (E2-ERα) signaling in ERα-positive breast cancer cells. In this study, we investigated the regulation of *CLOCK* expression by ERα and its roles in cell proliferation. Immunohistochemical analysis of human breast tumor samples revealed high expression of CLOCK in ERα-positive breast tumor samples. Subsequent experiments using ERα-positive human breast cancer cell lines showed that both protein and mRNA levels of CLOCK were up-regulated by E2 and ERα. In these cells, E2 promoted the binding of ERα to the EREs (estrogen-response elements) of *CLOCK* promoter, thereby up-regulating the transcription of *CLOCK*. Knockdown of CLOCK attenuated cell proliferation in ERα-positive breast cancer cells. Taken together, these results demonstrated that *CLOCK* could be an important gene that mediates cell proliferation in breast cancer cells.

## Introduction

Breast cancer is one of the most prevalent causes of cancer death among women. Prolonged exposure to estrogen is thought to be a major factor contributing to the development and progression of breast cancer [1], [2]. About 70% of breast cancers are estrogen-dependent. Moreover, clinical studies in which anti-estrogen or aromatase inhibitors are used to decrease the rate of local and distant relapse have demonstrated that estrogen can facilitate the progression of breast cancer [3].

The molecular mechanism of breast cancer induced by estrogen is thought to occur through the binding of estrogen to the transcription factor estrogen receptors (ERs), which then binds to estrogen response elements (EREs) in the promoters or regulatory regions of target genes. ERs contain two isoforms, ERα and ERβ, and each is encoded by a different gene. ERα is highly expressed in ER-positive breast cancer and is associated with breast cancer growth [1], [4], [5]. ERβ is also expressed in breast cancer, but its role is still elusive [6]. Moreover, ERα can bind to the promoter or regulatory regions of target genes that contain imperfect or truncated EREs, and activate their transcription [7], [8]. E2-ERα signaling plays a critical role in cell proliferation [9]. E2 promotes the proliferation of breast cancer cells through a number of established pathways [3].

Circadian rhythm is conserved across a wide range of organisms, including *Arabidopsis, Drosophila*, and mammals [10]. The duration of a circadian cycle is about 24 h. In mammals most physiological and behavioral functions are influenced by circadian rhythm. These rhythms are directed by endogenous clocks residing in the hypothalamic suprachiasmatic nucleus (SCN) and peripheral tissues [11], [12]. The molecular components of the circadian rhythm, clock genes and their products form the transcription-translation feedback loops. Two core transcription factors, CLOCK (circadian locomotors output cycles kaput) and BMAL1 (brain and muscle ARNT (aryl hydrocarbon receptor nuclear translocator)-like protein 1), form a heterodimer that binds to the E-box in the promoters of their target genes and activate the expression of these genes, including *Period* (*PER1, 2* and *3*) and *Cryptochrome* (*CRY1* and *2*). PER and CRY proteins can form heterodimer complexes that translocate to the nucleus, where they interferes with the transcriptional activity of BMAL1/CLOCK to limit their own expression, thereby constituting a negative-feedback loop [13], [14]. The circadian negative-feedback loop results in the circadian expression of clock genes.

There is accumulating evidence to suggest that circulating hormones could regulate the circadian oscillations of clock gene expression in some brain regions and peripheral tissues [15]–[18]. The ability of rhythmically-produced hormones to regulate the expression of clock genes in specific tissues implies a relationship between circadian clock and hormone production [12], [18]. Circulating levels of hormones can modulate circadian clock, which in turn regulates the periodic release of these hormones [19]. Recent reports have suggested that the circadian rhythm and the physiological condition of the body can mutually influence each other in mammals [20]. More and more evidence is suggesting that circadian disruption is associated with tumor occurrence, including breast cancer [21]–[23]. Estrogen plays a critical role in normal mammary gland physiology. At the same time it also acts as a potent mammary mitogen. Although the circadian clock is linked to the activity of estrogen, the molecular mechanisms underlying the regulation of the core clock genes that regulate the mammary circadian regulation are largely unknown.

It has been reported that upon treatment with E2, expression of the core clock gene *BMAL1* expression is enhanced [16]. Another circadian clock gene, *PER2,* is also a target gene of ERα that is regulated by E2 [18], [24], [25]. In rat uterus, E2 induces the high expression of *Per1* [15], [26]. These studies indicate that there is a relationship between E2-ERα signaling and the gears of circadian rhythm machinery. In our previous study, we have confirmed that CLOCK interacts with ERα and enhances its transcriptional activity [27]. Another breast cancer-associated protein, DEC1, has been shown to repress the transcriptional activity of CLOCK [28]. Many studies have focused on the transcriptional activity of CLOCK, but the transcriptional regulation of *CLOCK* is largely unknown until the revelation that the nuclear receptor REV-ERBα, a critical component of the circadian clock [29], is a transcriptional repressor of *CLOCK* [29], [30]. Based on the relationship between E2-ERα signaling and circadian clock genes, we wanted to know whether CLOCK is under the control of E2-ERα signaling.

In this study, we found that in ERα-positive breast cancer cells, E2 treatment increased while knockdown of ERα decreased the expression of *CLOCK*. In addition, we showed that ERα could bind to *CLOCK* via EREs and activate *CLOCK* transcription in response to E2. Taken together, our data suggested that *CLOCK* is a transcriptional target of ERα, and that the product of this gene can modulate cell proliferation in ERα-positive breast cancer cells.

## Material and Methods

### Ethics Statement

All research involving human participants have been approved by the institutional review board of Qiqihar Medical University. We have obtained the written consent from all the human participants and our clinical investigations have been conducted according to the principles expressed in the Declaration of Helsinki.

### Cell Culture and Experiment Reagents

Human breast cancer cell lines MCF-7, T47D, MCF10A and MDA-MB-231 have been used in our previous studies [31]–[34]. MCF-7 and MDA-MB-231 cells were cultured in Dulbecco's Modified Eagle's Medium (DMEM) containing 10% fetal bovine serum (Hyclone, Logan, UT), 100 µg/ml penicillin and 100 µg/ml streptomycin. T47D cells were cultured as previously described [35]. MCF10A were cultured in DMEM/F12 (1∶1) containing 5% horse serum (Hyclone, Logan, UT), 100 ng/ml Cholera Toxin, 10 µg/ml bovine insulin, 0.5 µg/ml hydrocortisones (sigma) and 20 ng/ml EGF (Peprotech, Rehovot Israel). Unless otherwise stated, all cell cultures were incubated at 37°C in the presence of 5% CO_2_. Cycloheximide (CHX), actinomycin D (Act D) and anti-FLAG antibody were obtained from Sigma. Anti-ERα, anti-HA, and anti-CLOCK were obtained from Santa Cruz Biotechnology (Santa Cruz, CA, USA), and 17β-estrogen (E2) and ICI 182780 (ICI) were obtained from Abcam (Cambridge, UK).

### Plasmids Constructions

The promoter region of human *CLOCK* (gene ID 9575) was amplified from a human genomic DNA by PCR and cloned into the plasmid pGL3-basic. Two truncated versions of *CLOCK* promoter were constructed, and each was fused to a luciferase reporter gene. CLOCK-WT-Luc (−884/+992) was amplified by sense primer 5′-GATCGGTACCCCAGTAGAAGCACTGAAATG-3′ and antisense primer 5′-GATCCTCGAGTCGCTGGAGTCAGACGCTAAT-3′; truncated CLOCK-M1-Luc (−297/+63) was amplified by sense primer 5′-GATCGGTACCAAAGCCAAAGAGCCTCC-3′ and antisense primer 5′-GATCCTCGAGTTTTAAACCGGCAGCC-3′; truncated CLOCK-M2-Luc (+552/+992) was amplified by sense primer 5′-GTACGGTACCGAGCTGCGGCCGATTCC-3′ and antisense primer 5′-GATCCTCGAGTCGCTGGAGTCAGACGCTAAT-3′. An ERE half-site and an ERE in CLOCK-WT-Luc were also mutated using a site-directed mutagenesis kit (Stratagene, La Jolla, CA, USA) according to the manufacturer's instruction. The mutant CLOCK-M3-Luc contained base substitutions in the ERE half-site of *CLOCK* (+2/+6), whereas the mutant CLOCK-M4-Luc contained base substitutions in the ERE of *CLOCK* (+753/+764), while the mutant CLOCK-M5-Luc contained base substitutions in both ERE half-site and ERE. The mutants were each generated by a pair of primer: CLOCK-M3-Luc (sense primer 5′-CCGCGGGGTCGCTTGCGACGCATGCGCCGG-3′, and antisense primer 5′-CCGGCGCATGCGTCGCAAGCGACCCCGCGG-3′), CLOCK-M4-Luc (sense primer 5′-CTGGGGACCCGCTAGGCAATGTTGCGCACTTTATTCCTGTCA-3′, antisense primer 5′-TGACAGGAATAAAGTGCGCAACATTGCCTAGCGGGTCCCCAG-3′). All cloned and mutated genes were verified by DNA sequencing. HA-REV-ERBα construct was a gift kindly provided by Dr. Hiromitsu Negoro (Kyoto University Graduate School of Medicine).

### Luciferase Reporter Assay

HeLa or MCF-7 cells were transfected with the appropriate plasmids, and 24 h after transfection, the cells were rinsed with PBS and subjected to luciferase activity assays. Briefly, the cells were lysed in cold buffer containing 25 mM glycylglycine (pH 7.8), 1% Triton X-100, 4 mM EGTA, 1 mM DTT, and 15 mM MgSO_4_. Five microliters of assay buffer (1 M MgCl_2_, 0.5 M KH_2_PO_4_, and 0.1 M ATP) and 100 µl 0.2 mM luciferin potassium salt (BD Biosciences Pharmingen, Franklin Lakes, NJ) were added to 45 µl cell lysate [36], and the luciferase activity of the sample was measured with a Centro LB 960 Microplate Luminometer (Berthold Techologies GmbH Co KG, Germany). The efficiency of transfection was evaluated by transfecting the cells with a β-galactosidase construct. Briefly, 20 µl cell lysate was added to 50 µl β-galactosidase buffer (60 mM Na_2_HPO_4_·12H_2_O, 40 mM NaH_2_PO_4_·2H_2_O, 10 mM KCl, 1 mM MgSO_4_·7H_2_O, and 6 mg/ml ONPG) and the absorbance of the sample was measured at 450 nm.

### RNA Extract and RT-PCR

Triozol reagent (Invitrogen, Grand Island, NY) was used to extract the total RNA from MCF-7 cells. The extraction was performed according to the manufacturer's instruction and the concentration of RNA was quantified by optical density. One microgram of total RNA was retrotranscribed into cDNA using Reverse Transcription System (TAKARA, Dalian, China). Real-time PCR was performed with a Roter-Gene 3000 (Corbett Research, Australia) using the following primers as reported: 5′-AAGTTAGGGCTGAAAGACGACGA-3′ (sense) and 5′-GAACTCCGAGAAGAGGCAGAAG-3′ (antisense) for *CLOCK* [16]; 5′-GAAGGTGAAGGTCGGAGTC-3′ (sense) and 5′-GAAGATGGTGATGGGATTTC-3′ (antisense) for *GAPDH* [16]. The cDNA was combined with the appropriate pair of primers and the maxima SYBR Green qPCR Master Mix (Thermo scientific) and subjected to the following reaction: initial denaturation step of 95°C for 10 min; and 40 cycles of 95°C for 20 s; 56°C for 20 s and 72°C for 20 s. The efficiency of the real-time PCR assay was determined from the amplification efficiency E and linear correlation coefficient R^2^. Ten-fold serial dilutions (from 10^−6^ to 10^−2^) of cDNA generated from MCF-7 cells were used in the real-time PCR assay to generate a set of data for the standard curve. E and R^2^ values were calculated from the standard curve as in previous report [37]. To evaluate the quality of the product of real-time PCR, melt curve analyses were performed after each reaction. *GAPDH* is a frequently used housekeeping gene in real-time PCR as it is expressed at a relatively constant level in various tissues, including breast tissue, under normal and pathophysiological conditions [38]–[40]. Therefore, the expression level of *CLOCK* was normalized the expression level of *GAPDH* using Roter Gene 6.0 software. Relative expression was determined using the 2^−ΔΔCt^ method with *GAPDH* as the reference gene. Each target was measured in triplicate.

### Western Blot Analysis

Preparation of cell extracts and subsequent western blot analysis were carried out as previously described [28]. Immunoblot data were quantified by scanning the appropriate bands of interest and plotted as relative density of gray scale.

### RNA Interference

ERα shRNA-expression vector was constructed by DNA vector-based shRNA synthesis using the vector pRNATU6.1 (GenScript, Piscataway, NJ). The sequence of ERα used for knockdown study were 5′-GCTACTGTTTGCTCCTAAC-3′ (shERα#1) [41] and 5′-AGTTTGTGTGCCTCAAATC-3′ (shERα#2) [42]. The sequences used for silencing the expression of *CLOCK* have been described in our previous study [27]; and the sequence of the control shRNA is 5′-GACGCTTACCGATTCAGAA-3′ [35], which has no significant homology with human gene sequence. shERα#1 and shERα#2 expression vectors were verified by DNA sequencing.

### Chromatin Immunoprecipitation Assays

MCF-7 cells were grown for 2 days in phenol red-free DMEM containing 5% charcoal-dextran-stripped FBS. The cells were then treated with or without 1 µM E2 for 1 h, and then crosslinked with 1% formaldehyde in PBS for 15 min at room temperature. Crude cell lysate was sonicated to generate DNA fragments of 300 to 1500 bp. The generated DNA fragments were diluted 1∶10 in dilution buffer (150 mM NaCl, 2 mM EDTA, 1% Triton X-100, and 20 mM Tris-HCl pH 8.0) [35]. Protein A and anti-ERα antibody or rabbit IgG were then added to the diluted sheared chromatin, and the mixture was incubated with constant rotation at 4°C for overnight. The immunoprecipitated chromatin was purified from the chromatin-antibody mixture and eluted in elution buffer (50 mM Tris-HCl pH 8.0, 10 mM EDTA, and 10% SDS). The isolated DNA was subjected to PCR to amplify the regions using specific primers: 5′-GAGCTGCGGCCGATTCC-3′ (sense) and 5′-GCTGCTCCAAACGTGC-3′ (antisense) for *CLOCK* (+672/+805); 5′-AAAGCCAAAGAGCCTCC-3′ (sense) and 5′-TTTTAAACCGGCAGCC-3′ (antisense) for *CLOCK* (–297/+63); and 5′-TGAAAGAGGGAGGAGTCAAAGAT-3′ (sense) and 5′-AGCAAGACGGAGGCAAAGTTATT-3′ (antisense) for *CLOCK* (–1866/–1626). Total input DNA (1∶10 dilution) was used as a positive control for the PCR reaction. The anti-IgG antibody was used as a non-specific binding control. The PCR products were analyzed by electrophoresis using 1.5% agarose gel.

### Cell Proliferation Assays

Cell proliferation was assessed by MTT assay. MCF-7 or T47D cells were cultured for 24 h in phenol red-free DMEM supplemented with 5% charcoal-dextran-treated fetal bovine serum. The cells were then transfected with shControl (shCon), shCLOCK or shERα#1 construct. After 24 h, the cells were treated with vehicle or 1 µM E2 for several days, and then subjected to MTT assay performed with a commercial kit (Key Gen) according to the manufacturer's protocol. The absorbance of the samples was read at 490 nm [27]. For colony formation assays, MCF-7 cells were transfected with shControl (shCon), shCLOCK or shERα#1 construct, and the cells were then collected and plated at a density of 1000 cells/well in 24-well plates, and treated with vehicle (ethanol) or 1 µM E2 for seven days. After that the cells were washed with PBS, fixed with ethanol, stained with 0.1% crystal violet, and then photographed. The stained cells were solubilized in 10% SDS, and absorbance was measured at 570 nm [43].

### Soft-Agar Colony Culture

The anchorage-independent growth of MCF-7 cells was estimated by soft-agar colony culture as described previously [44], [45]. MCF-7 cells were transfected with CLOCK expression vector, ERα expression vector or empty vector (pcDNA3) and grown in the presence of 1 µg/ml G418 for 2 weeks. Aliquot of the cell suspension containing 1000 cells was mixed with 1 ml DMEM containing 10% fetal bovine serum and 0.4% agar, and then poured over a layer of solidified 0.7% agar (prepared in 1 ml medium) in a well of a 6-well plate. Additional 500 µl DMEM containing 10% fetal bovine serum was added to the well every two days. One week after seeding, photographs of the colonies were taken under phase-contrast microcopy, and the diameters of the colonies were measured by the software Image-Pro Plus 6.0 [46].

### Breast tumor tissue samples for immunohistochemical assay

Breast tumor samples used for immunohistochemical assay were obtained from Qiqihar Medical University. The specimens were obtained from female patients of Han Chinese descent, with ages ranging from 39 to 75 years old (average age of 56.7 years). A total of 32 specimens were obtained and 19 of these were ERα-positive and 13 were ERα-negative, as determined by clinical diagnosis performed by Qiqihar Medical University. The tumor grades were recorded as II (22 specimens), III (3 specimens) or II-III for obscure tumor grade (7 specimens).

### Immunohistochemical Assay

All the obtained human breast tumor specimens were analyzed by immunohistochemical assay, which was performed with the aid of an immunohistochemical assay kit (Maixin Bio, China). Sections of the tissues were first fixed in 10% buffered formalin. The fixed tissues were embedded in paraffin and then deparafinninized before being rehydrated using standard procedures [47]. The endogenous peroxidases of the samples were quenched with H_2_O_2_ in methanol. After that, the samples were incubated in blocking solution (4% bovine serum albumin, 0.1% Triton X-100, 0.1 M PBS) for 10 min [48] and then stained with citrate buffer (pH 6.0) containing a 1∶100 dilution of either rabbit anti-human CLOCK (Santa Cruz, CA, USA) or rabbit anti-human ERα (Santa Cruz, CA, USA) for overnight at 4°C. Following incubation with primary antibody, the slides were then incubated with a 1∶200 dilution of biotinylated goat anti-rabbit IgG for 10 minutes at room temperature, and then incubated in Avidin-Biotin Peroxidase Complex for 10 minutes at room temperature. After washing for three times in PBS they were incubated with DAB (diaminobenzidine), which was used as chromagen for the antibody. The intensity of the staining, which reflected the level of CLOCK/ERα in the sample, was quantified by assigning it an H score. The procedure for acquiring an H score was performed as described previously [49], [50]. The levels of CLOCK expression in these breast tumor samples were therefore sorted according to their H scores.

### Statistical Analysis

A Chi-square (*χ* ^2^) test was used to examine the correlation between CLOCK and ERα gene expression in breast cancer tissues from 32 patients. All other data were expressed as means ± SDs. Differences between mean values were analysed by ANOVA, followed by the Bonferroni test for pairwise comparisons. Statistical significance was considered at the *P*<0.05 level.

## Results

### CLOCK protein is upregulated in ERα-positive breast tumor

**Figure 1:**
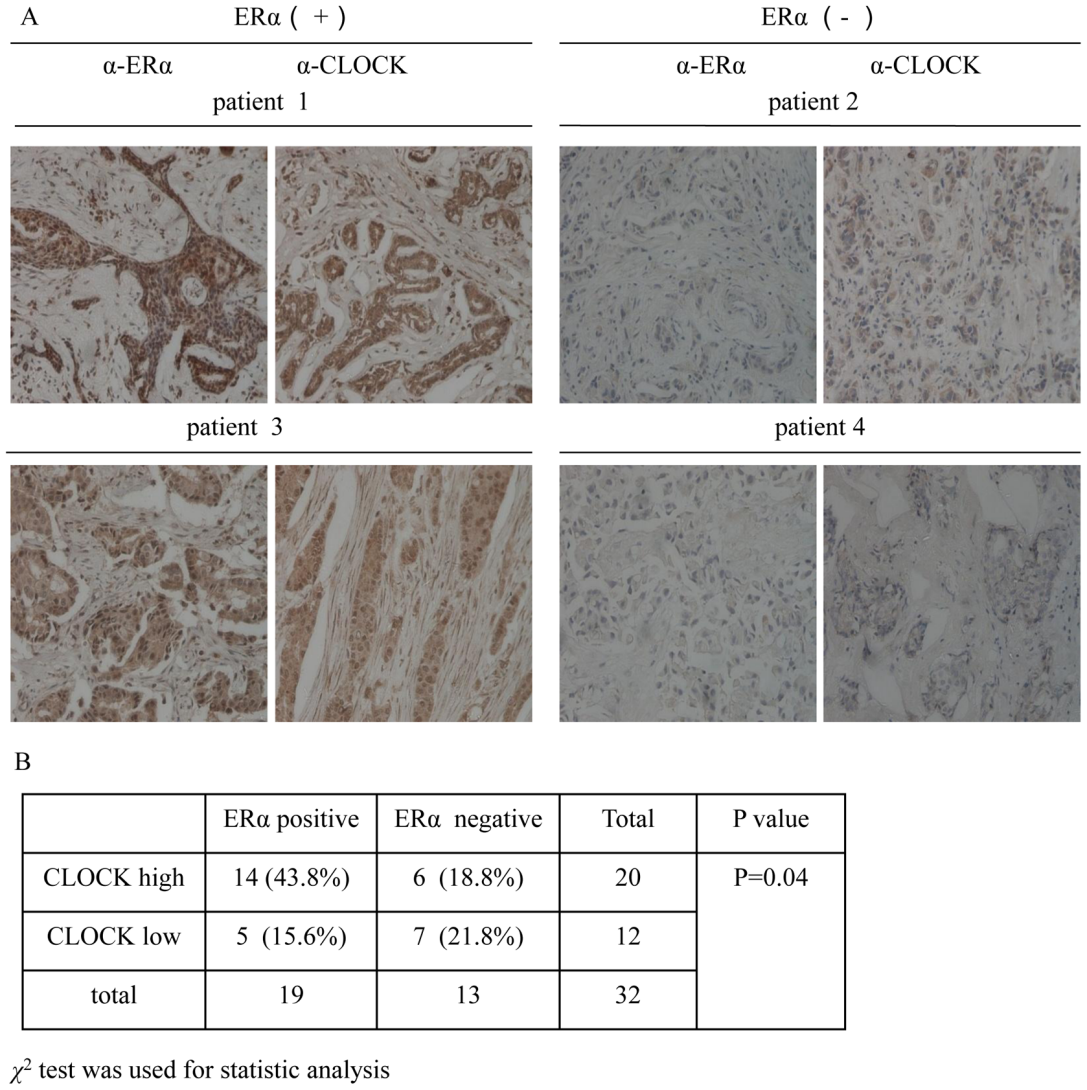
Correlation between ERα and CLOCK expression in human breast tumor tissue samples. A, Representative results showing the immunohistochemical staining of ERα and CLOCK in serial sections of the breast tumor tissues. Each sample was incubated with antibody against ERα or CLOCK. Positive staining and negative staining are indicated by brown and blue staining, respectively (×200 Magnification). B, Correlation between ERα and CLOCK expression suggested by the 32 breast tumor samples. *χ* ^2^ test was used for statistical analysis. *P* values less than 0.05 were considered to indicate statistical significance.

It has been reported that aberrant ERα signaling is related to the occurrence of ERα-positive breast tumor. ERα-positive breast tumor generally has a better prognosis, and is responsive to anti-estrogen therapy. However, the role of CLOCK in breast tumor has not been elucidated. In order to examine the relationship between CLOCK and ERα in breast tumor, we compared the protein levels of CLOCK and ERα in ERα-positive breast tumor samples with those of ERα-negative breast tumor samples (Fig. 1A). A total of 32 tissue samples (19 ERα-positive and 13 ERα-negative) were analyzed by immunohistochemical assay. Fourteen of the ERα-positive samples showed high CLOCK expression (74%). As for the 13 ERα-negative samples, high CLOCK expression was found in only 6 samples (46%) (Fig. 1B). The data appeared to suggest a correlation between the ERα and CLOCK in ERα-positive breast tumors.

### ERα regulates the level of CLOCK protein

**Figure 2:**
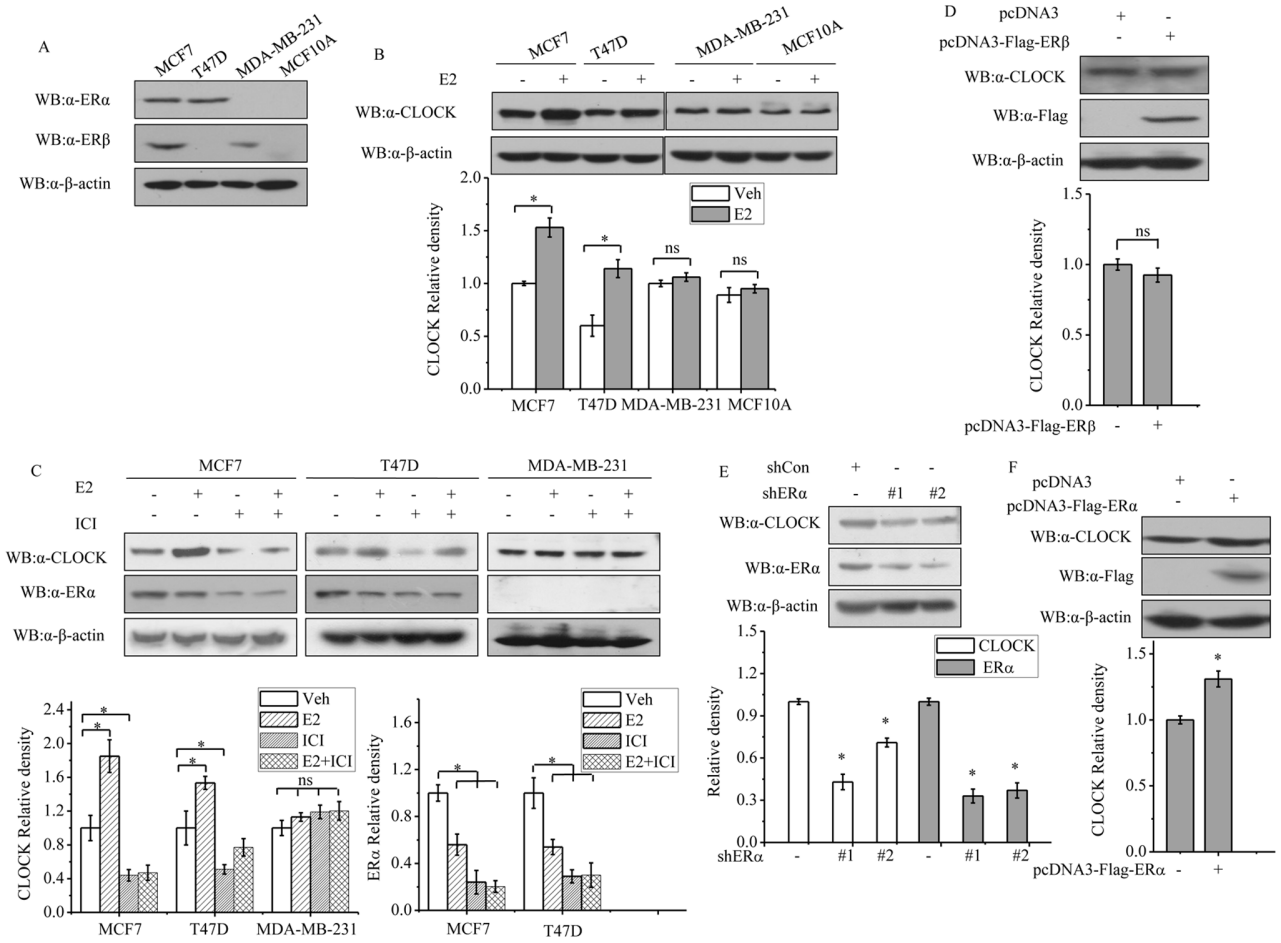
Western blot analyses of CLOCK and ERα expression in cells treated with E2 or ICI. A, ERα and ERβ expression in MCF-7, T47D, MDA-MB-231, and MCF10A cells. B, CLOCK expression in MCF-7, T47D, MDA-MB-231, and MCF10A cells that had been treated with vehicle (control) or 1 µM E2 for 24 h. Cells were cultured in 5% charcoal striped FCS and phenol red free medium for 2 days before stimulated with E2. C, CLOCK and ERα expression in MCF-7, T47D and MDA-MB-231 cells that had been treated with vehicle, 1 µM E2 or 0.1 µM ICI alone or in combination for 24 h. Cells were cultured for 2 days in 5% charcoal striped FCS and phenol red free medium for two days before they were treated with ER ligands. D, CLOCK and ERα expression in T47D cells transfected with empty vector pcDNA3 or pcDNA3-Flag-ERβ. E, CLOCK and ERα expression in MCF-7 cells transfected with control shCon or two different shERα (shERα#1 and shERα#2). F, CLOCK expression in MCF-7 cells transfected with pcDNA3 or pcDNA3-Flag-ERα. B-F, 24 h after transfection, the cells were harvested and subjected to western blot analysis. In all experiments (A-F), β-actin expression was used as a reference. The blot shown is the representative result from three independent experiments. Image of the blot is shown in the top panel of each figure, with the quantitative analysis of the bands in the blot shown in the plot below. The levels of CLOCK or ERα signal obtained from control cells were set to 1. All experiments were repeated at least three times. Data shown in the graphs are the means ± SDs of three experiments. *P* value was determined by ANOVA with Bonferroni test (*, *P*<0.05. ns, not significant).

Circulating estrogen (E2) is known to modulate the expression of some clock genes. Given the apparent correlation between ERα and CLOCK expression in the breast-tumor samples analyzed, we next examined whether CLOCK expression could be stimulated by E2. Treatment of the ERα-positive breast cancer cell lines, MCF-7 and T47D cells with 1 µM E2 for 24 h resulted in increased expression of CLOCK protein in these cells, but the same treatment given to the ERα-negative cell lines, MDA-MB-231 and MCF10A resulted in no apparent effect on CLOCK expression (Figs. 2A&B). The importance of ERα with respect to increased expression of CLOCK when MCF-7 and T47D cells were treated with E2 was further investigated by observing the change in CLOCK expression when these cells were treated with the anti-estrogen agent, ICI182780 (ICI) instead of E2. ICI treatment resulted in a reduction of CLOCK expression. Furthermore, the E2-enhanced expression of CLOCK in these cells was partly reversed when the same cells were also treated with ICI (Fig. 2C). The level of ERα in these cells was also down-regulated after treatment with E2 or ICI, which was consistent with previous reports [51]. In contrast, MDA-MB-231 cells, which are ERα-negative but ERβ-positive, showed no obvious changes in the level of CLOCK expression when treated with E2 or ICI (Fig. 2C). T47D is an ERα-positive but ERβ-negative breast cancer cell line. Overexpression of ERβ in T47D cells had no profound impact on the level of CLOCK (Fig. 2D). These results suggested that ERβ may have a minimal effect on the expression of CLOCK. To determine whether E2 could stimulate the expression of CLOCK through ERα, the expression of ERα was knocked down with two different shRNAs, shERα#1 and shERα#2, which target different regions of the ERα mRNA to avoid possible off-target effect. The effectiveness of the two shRNAs has already been demonstrated by other investigators [41], [42]. Knockdown of ERα decreased the expression of CLOCK (Fig. 2E). When ERα was overexpressed in MCF-7 cells, the level of CLOCK protein increased slightly (Fig. 2F). Taken together, the results showed that expression of CLOCK is subject to control by ERα.

### ERα modulates the transcription of *CLOCK*

**Figure 3:**
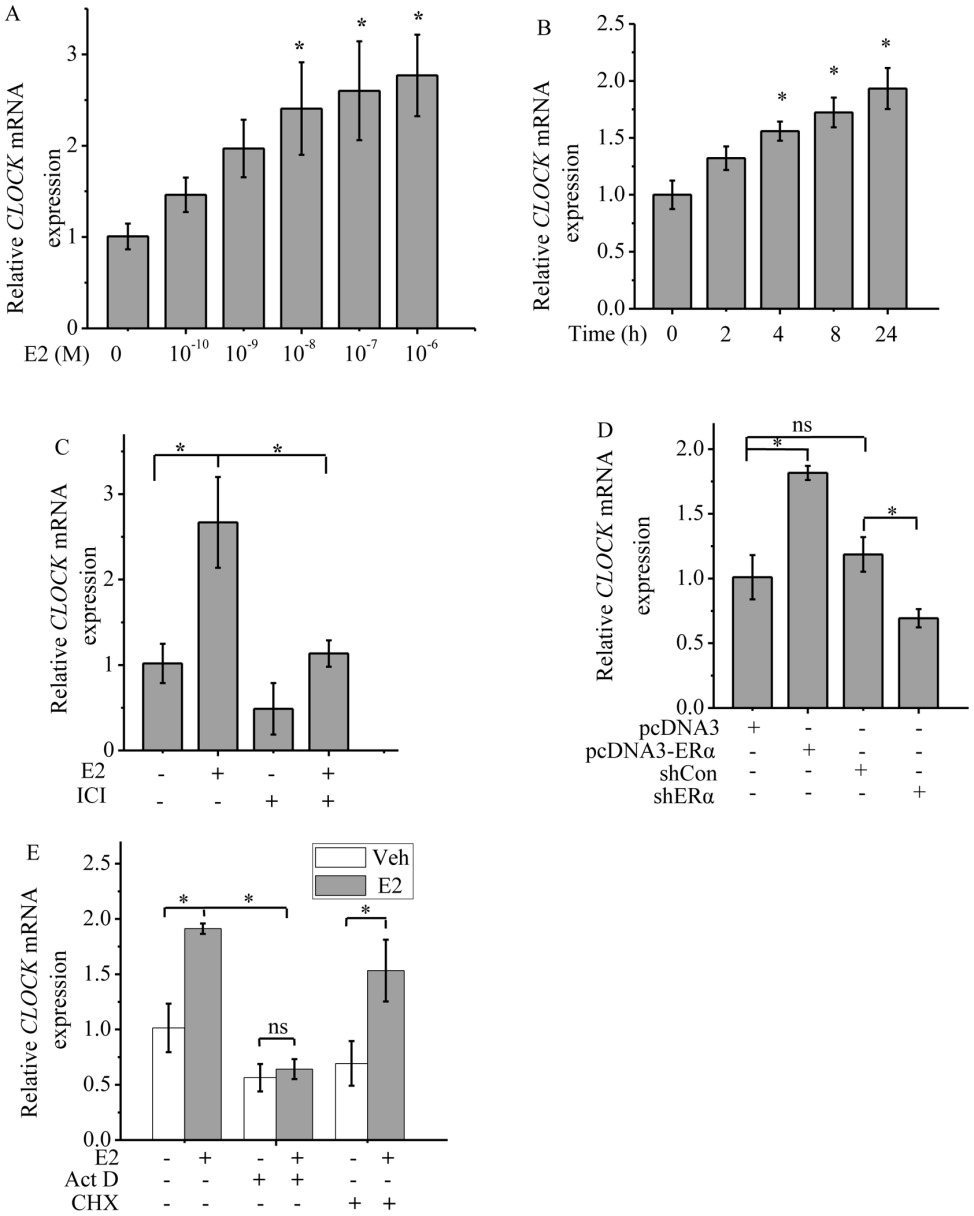
ERα ligands regulate the expression of CLOCK at the transcription level. Analysis of CLOCK mRNA levels in MCF-7 cells by real-time PCR. MCF-7 cells were cultured in phenol red free medium and charcoal striped FCS medium for 2 days before being treated with E2 or ICI and the expression of *CLOCK* was then analyzed by real-time PCR. Expression of *CLOCK* was normalized against *GAPDH* mRNA level (internal control). A, Cells treated with different concentrations of E2 (10^−10^ to 10^−6^ M) for 8 h. B, Cells treated with 1 µM E2 for different periods of time. C, Cells treated with 1 µM E2 or 0.1 µM ICI for 12 h. D, MCF-7 cells transfected with empty vector for ERα (pcDNA3), ERα, shCon (control for shERα) or shERα#1 construct. E, MCF-7 cells were cultured in phenol red-free medium and charcoal-striped FCS medium for 2 days before being treated with E2, Act D or CHX and the expression of *CLOCK* was then analyzed by real-time PCR. Cells treated with 0.5 µg/ml Act D, 10 µg/ml CHX alone or in combination with 1 µM E2 for 12 h. A-E, Relative levels were calculated by giving an arbitrary value of 1 to the control. *CLOCK* transcript levels were normalized to *GAPDH* transcript level and expressed as arbitrary units relative to the vehicle control (set as 1). Each experiment was performed in triplicate and repeated at least three times. Data shown are the means ± SDs. *P* value was determined by ANOVA with Bonferroni test (*, *P*<0.05. ns, not significant).

The effect of ERα on the level of CLOCK expression was further investigated by determining the changes in the level of *CLOCK* mRNA in MCF-7 cells in response to E2 or ICI after 24 h of treatment. *CLOCK* mRNA level was up-regulated in response to E2 in a dose-dependent manner in the range of 10^−10^ to 10^−6^ M (Fig. 3A). Thus 10^−6^ M E2 was chosen for subsequent studies. *CLOCK* mRNA level was increased 4 h after E2 treatment (Fig. 3B). However, when the cells were treated with ICI, the level of *CLOCK* mRNA was reduced compared to that of the control (Fig. 3C). The effect of ERα on the modulation of *CLOCK* transcription in response to E2 was further confirmed by overexpressing ERα in MCF-7 cells and knocking down ERα with shERα. As expected, ERα ectopic expression up-regulated *CLOCK* transcription, while knockdown of ERα down-regulated *CLOCK* transcription (Fig. 3D). In addition, Act D repressed the basal expression of *CLOCK* and abolished E2-induced up-regulation of *CLOCK*. Although Act D globally represses gene transcription, including the transcription of *GAPDH*, it inhibited the transcription of *CLOCK* more than that of *GAPDH*. In contrast, cycloheximide (CHX, a translation inhibitor) had no effect on E2-induced up-regulation of *CLOCK* (Fig. 3E), indicating that *CLOCK* is a primary ERα transcriptional target because the effect of E2 does not require the synthesis of new proteins since all necessary factors are preexisting in the cells.

### ERα regulates *CLOCK* promoter activity

**Figure 4:**
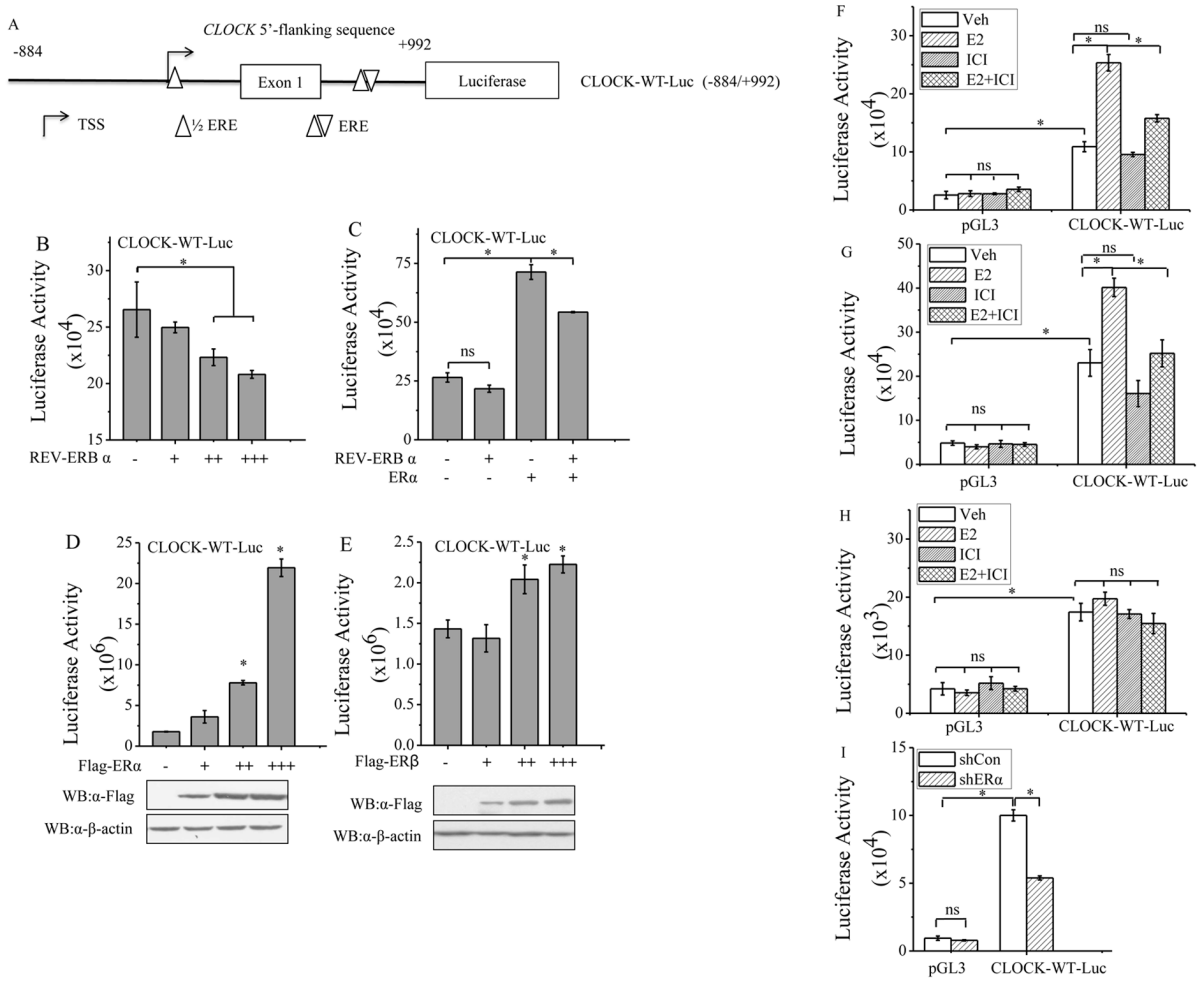
ERα regulates *CLOCK* promoter activity. A, Schematic illustration of estrogen response elements in *CLOCK* promoter containing *CLOCK* sequence from −884 to +992 fused to luciferase (CLOCK-WT-Luc). B, Luciferase activity of HeLa cells transfected with CLOCK-WT-Luc and increasing amounts of REV-ERBα expression plasmid. C, Luciferase activity of HeLa cells transfected with CLOCK-WT-Luc plus REV-ERBα or ERα expression plasmids or both. D, Luciferase activity of HeLa cells transfected with CLOCK-WT-Luc and different amounts of ERα expression plasmid. E, Luciferase activity of HeLa cells transfected with CLOCK-WT-Luc and increasing amounts of ERβ expression plasmid. MCF-7 (F), T47D (G) and MDA-MB-231 (H) cells were grown in steroid-depleted media for 2 days, and then transfected with CLOCK-WT-Luc, followed by treatment with E2 or ICI alone or in combination. F-H, For control, cells were transfected with pGL3. pGL3 containing no *CLOCK* sequence was used as a mock DNA constructs. I, MCF-7 cells grown in normal media were transfected with the indicated shRNA (ERα; Con as a negative control) and CLOCK-WT-Luc. B-I, The graph depicts the normalized luciferase activity for each condition. Each experiment was performed in triplicate and repeated at least of three times. Data shown are the means ± SDs. *P* value was determined by ANOVA with Bonferroni test (*, *P*<0.05. ns, not significant).

The transcription of CLOCK has been shown to be repressed by nuclear receptor REV-ERBα through its interaction with the REV-ERB response element (RevRE) located 760–771 bases downstream the transcription start site (TSS) of the *CLOCK* gene [30]. As the location of the regulatory element is in the first intron of the *CLOCK* gene, a 1877-bp fragment encompassing the regions upstream and downstream of the TSS was cloned (See Fig. 4A). This fragment which included the reported RevRE was fused to the luciferase gene, generating the construct CLOCK-WT-Luc. HeLa cells transfected with CLOCK-WT-Luc and REV-ERBα showed a decreasing trend in luciferase activity that was dependent on the dosage of REV-ERBα (Fig. 4B), consistent with former report. However, overexpression of ERα could attenuate the repression of luciferase activity by REV-ERBα (Fig. 4C). HeLa cells were used because these cells have no detectable levels of ERα or ERβ. The cells were transfected with increasing amounts of ERα-expression plasmids and CLOCK-WT-Luc exhibited enhanced expression of luciferase activity that paralleled with the dosage of ERα gene (Fig. 4D). HeLa cells transfected with CLOCK-WT-Luc plus increasing amounts of ERβ resulted in minimal increase in luciferase activity compared to cells transfected with CLOCK-WT-Luc and ERα (Fig. 4E). To determine whether ERα could stimulate *CLOCK* transcription, the luciferase activity of MCF-7 cells transfected with CLOCK-WT-Luc followed by treatment with E2 or ICI was determined. The level of CLOCK-WT-Luc luciferase activity was increased by 3.8-fold in the presence of E2, but such an enhancement was attenuated by ICI (Fig. 4F). In another breast cancer cell line T47D, similar results were obtained (Fig. 4G), but in MDA-MB-231 cells, the level of luciferase activity expressed by CLOCK-WT-Luc did not change significantly in response to E2 or ICI (Fig. 4H). As for MCF-7 cells, knockdown of ERα with shERα resulted in decreased level of reporter activity (Fig. 4I). These results confirmed that *CLOCK* was transcriptionally regulated by ERα.

### ERα bounds to *CLOCK* promoter regions in response to E2

**Figure 5:**
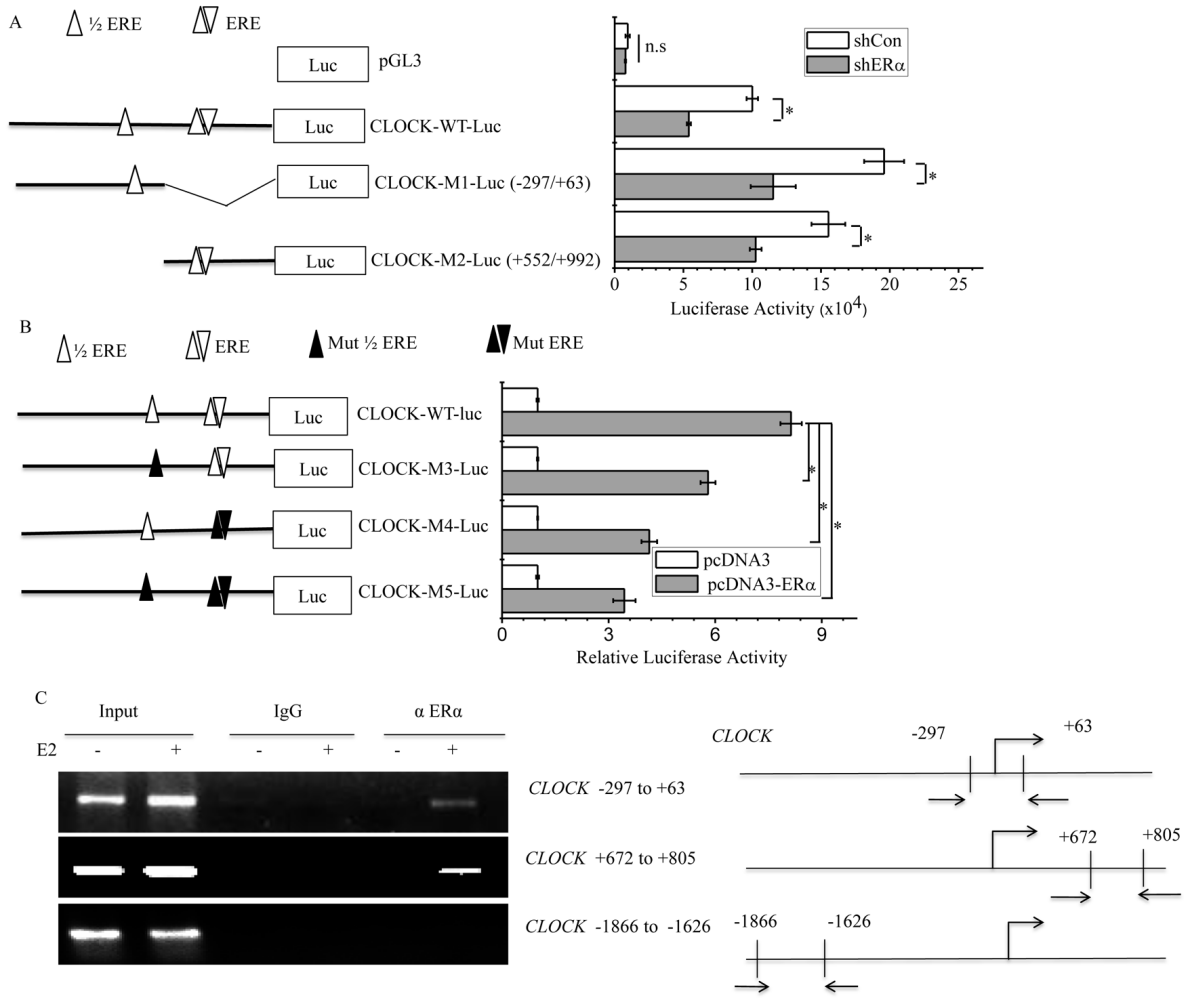
ERα bounds to *CLOCK* promoter regions in response to E2. A, Schematic representation of the ERE sites within the *CLOCK* promoter regions in the CLOCK-WT-Luc constructs. Constructs containing wild-type promoter and mutant promoters (truncation) are shown. Luciferase activity of MCF-7 cells transfected with the indicated constructs together with or without shERα#1 are shown on the right. B, CLOCK luciferase reporter constructs containing wild-type and mutant CLOCK promoters with point mutation in the EREs are shown, together with the luciferase activity of HeLa cells transfected with one of these constructs together with or without ERα. A and B, all experiments were performed in triplicate and repeated at least three times, and the data shown are the means ± SDs. *P* value was determined by ANOVA with Bonferroni test (*, *P*<0.05. ns, not significant). C, ChIP assay showing the recruitment of ERα on *CLOCK* promoter regions. MCF-7 cells were grown in phenol red-free medium and charcoal striped FCS medium for 2 days and the cells were then treated with vehicle or 1 µM E2 concentrations for 1 h, followed by ChIP assay using antibody against ERα or IgG. Total input DNA at a 1∶10 dilution was used as a positive control for the PCR reaction. Immunoprecipitated DNA was analyzed by PCR with primers specific for *CLOCK*, the relative positions of which are shown in the right panel of Figure 5C. All experiments were repeated at least of three times.

To map the ERα responsive regions within the *CLOCK* promoter region, a computational analysis was performed and the results indicated that half estrogen response element (^1^/_2_ERE) was present at the +2 to +6 region while an ERE was present at the +753 to +764 region. To delineate which portion of the promoter was responsive to ERα, two truncated versions of the promoter-fused luciferase were constructed, CLOCK-M1-Luc (−297/+63) and CLOCK-M2-Luc (+552/+992), and their activity in response to ERα was tested. MCF-7 cells transfected with either construct showed a decreased level of luciferase activity when ERα was knocked down, a trend that was also exhibited by wide-type CLOCK-WT-Luc (Fig. 5A). In a different experiment, constructs of CLOCK luciferase reporter bearing point mutation in the CLOCK component were also made. The point mutation consisted of two nucleotide substitutions at either the half ERE (CLOCK-M3-Luc), six nucleotide substitutions at the ERE site (CLOCK-M4-Luc) or both (CLOCK-M5-Luc). HeLa cells transfected with the wild-type construct and those transfected with any of the three mutant forms showed similar levels of luciferase activity in the absence of ERα overexpression. With ERα overexpression, the level of luciferase activity increased by about eight fold in the case of wild-type construct, about six fold for CLOCK-M3-Luc, four fold for CLOCK-M4-Luc and three fold for CLOCK-M5-Luc (Fig. 5B), indicating that although the presence of intact ERE was important for CLOCK-driven luciferase activity, such activity was dependent on the presence of ERα, which interacted with the ERE of the CLOCK promoter region. It was worth noting that ERα still activated the activity of CLOCK-M5-Luc despite the absence of any ERE (Fig. 5B). This could possibly be due to ERα interacting with other transcription factors that could bind to other regions of the *CLOCK* promoter in CLOCK-M5-Luc [52], [53]. Such binding would effectively enable ERα to bind to CLOCK-M5-Luc indirectly and consequently activate the transcription of the reporter gene. Surprisingly, in the case of nucleotide-substitution mutation, the result appeared to indicate that interaction between ERα and ERE was more important than between ERα and the half ERE, whereas in the case of truncation mutation, the result seemed to indicate the opposite. Nevertheless, both did confirm that ERE was essential for ERα-mediated upregulation of CLOCK activity.

We further tested the binding of ERα on the *CLOCK* gene in MCF-7 cells by chromatin immunoprecipitation (ChIP) assay *in vivo*. Following treatment of MCF-7 cells with E2, the DNA of the cells was immunoprecipitated and analyzed by PCR using the *CLOCK* gene specific primers correspond to regions −297 to +63, +672 to +805, or −1866 to −1626. ERα only bound to regions −297 to +63 and +672 to +805, but not region −1866 to −1626 in the presence of E2 (Fig. 5C). Regions −297 to +63 and +672 to +805 contains the ^1^/_2_ERE and ERE, respectively (left panel of Fig. 5A). These results suggested that the binding of endogenous ERα to the *CLOCK* gene is dependent on E2. ChIP results further supported the involvement of ERα in the transcription of *CLOCK* gene.

### CLOCK is required for the proliferation of breast cancer cells

**Figure 6:**
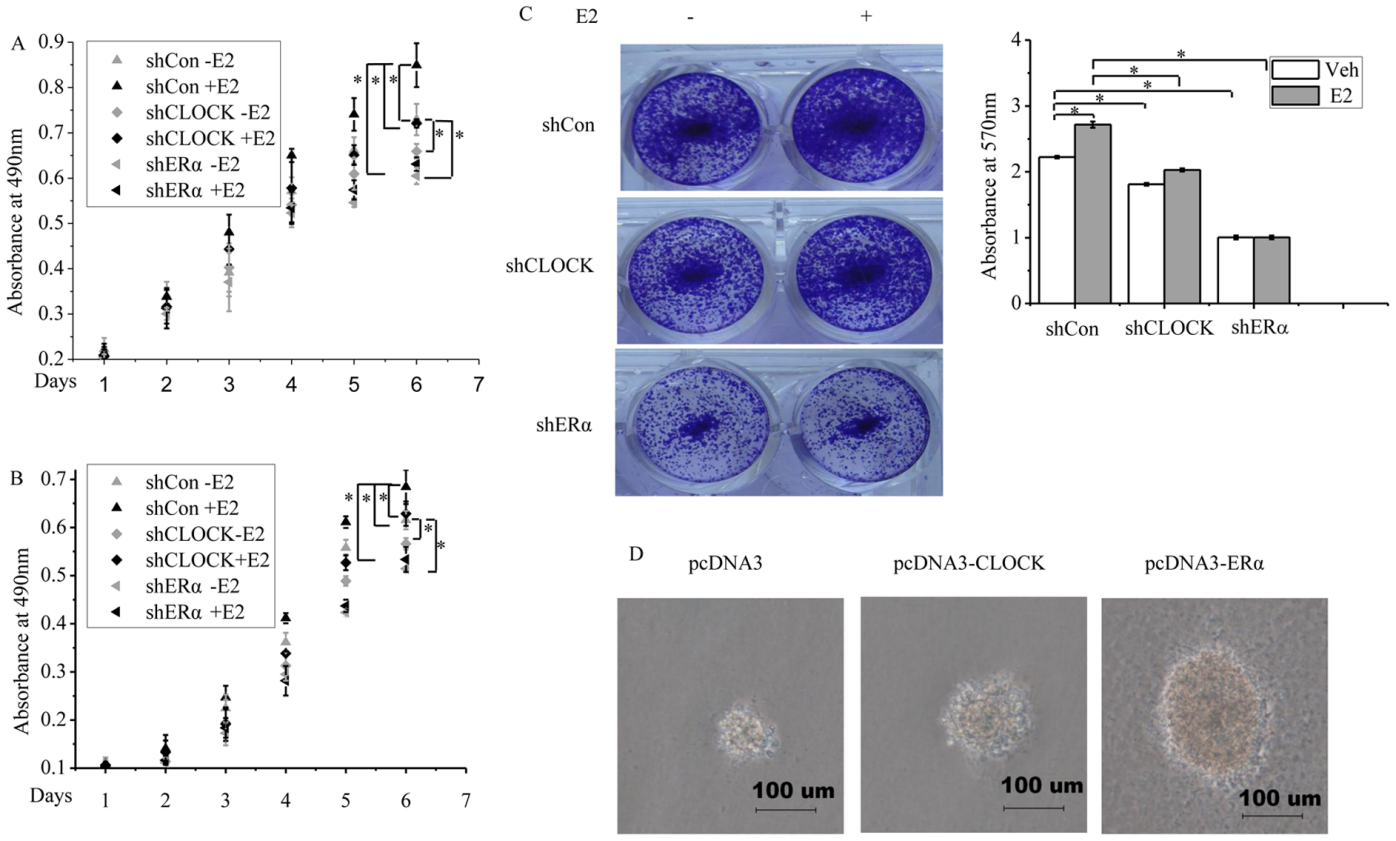
CLOCK promotes MCF-7 cells proliferation. Cells were transfected with control shRNA (shCon), shCLOCK or shERα#1 in the presence or absence of E2 for six or seven days followed by MTT assay or crystal violet staining. MTT assay of MCF-7 (A) and T47D (B) cells. The cells were treated with E2 for six days. C, Crystal violet staining (MCF-7 cells). The cells were treated with E2 for seven days. Viable colonies were stained with 0.1% crystal violet and photographed. The dye taken up by the colonies were solubilized in 10% SDS and quantified by absorbance at 570 nm. Representative images are shown on the left panel of Figure 6C, and the corresponding quantitative analyses are shown on the right panel. Only representative data from three independent experiments are shown. D, Representative colonies of each experimental group are shown. MCF-7 cells transfected with pcDNA3, pcDNA3-CLOCK or pcDNA3-ERα were selected in the presence of 1 µg/ml G418 for 2 weeks. The cells were then collected and subjected to a soft agar colony culture. Photographs of the colonies were taken one week after seeding. All experiments were repeated at least three times. A-C, Data are the means ± SDs. *P* value was determined by ANOVA with Bonferroni test (*, *P*<0.05).

The potential relevance of our findings to the biology of breast cancer cells was investigated by looking at the effect of reduced CLOCK expression on cell proliferation. E2 treatment stimulated the proliferation of MCF-7 cells transfected with shControl (Fig. 6A). In contrast, knockdown of CLOCK or ERα inhibited cell proliferation in the absence and presence of E2 (Fig. 6A). Similar results were obtained for T47D cells (Fig. 6B). These results suggested that CLOCK and ERα could promote cell proliferation. We next examined the effect of CLOCK knockdown on the colony formation of MCF-7 cells. E2 treatment increased the colony formation of MCF-7 cells (top panel of Fig. 6C). Knockdown of CLOCK decreased the colony formation of MCF-7 cells in the absence and presence of E2 compared to shControl (Fig. 6C). Knockdown of ERα expression also decreased the colony formation ability of the cells as expected, and at the same time the cells lost the response to E2 (bottom panel of Fig. 6C). Three-dimensional cell culture is considered superior to monolayer cultures because the growth of a cell in a three-dimensional culture resembles more the growth in an *in vivo* environment. Soft-agar culture was chosen to test the difference in growth among the MCF-7 cells transfected with different vectors (empty vector or vector harboring CLOCK or ERα gene). Cells that overexpressed CLOCK or ERα showed more growth than the cells that were transfected with the empty vector (Fig. 6D). These results indicated that the induction of *CLOCK* expression via ERα appears to constitute a driving force in the proliferation of ERα-positive breast cancer cells.

## Discussion

Apparently, women who have been exposed to artificial light at night for long term, or have been working with jobs that can alter their circadian rhythm are predisposed to breast cancer [54]–[56]. Growing evidence suggests that breast tumorigenesis is associated with the disruption of circadian clocks [23], [56], [57]. Thus it is important to investigate how estrogen signaling, which is vital to a number of cellular processes and the onset of breast cancer, is integrated with the circadian clock. CLOCK is a core transcription factor in the transcription-translation feedback loops of the machinery that regulates circadian rhythm. However, the molecular details of the transcriptional regulation of *CLOCK* remain largely unknown. In this study we demonstrated that *CLOCK*, similar to other circadian clock genes, is subject to modulation by estrogen in breast cancer cells.

A higher percentage of ERα-positive breast tumor samples that we analyzed revealed a high level of CLOCK protein compared to ERα-negative breast tumor samples (74% versus 46%, Fig.1B), which suggested that the transcription of CLOCK in ERα-positive tumor may be upregulated. However, there appeared to be no correlation between the expression of CLOCK and ages. Due to the obscure tumor grades recorded for some of the specimens, it was not possible to establish a correlation between CLOCK expression and tumor grades. The time of tumor resection may be important for the study of circadian clock proteins, but in our study it was not thoroughly recorded. To our knowledge, differences in the expression levels of breast-tissue CLOCK protein between day and night, or changes in the expression levels during menstruation have not yet been reported. Although the number of breast tumor samples analyzed may be low, the correlation between CLOCK and ERα was statistically significant and should not be overlooked as a chance event, as subsequent experiments employing two ERα-positive breast carcinoma cell lines, MCF-7 and T47D, revealed that the levels of CLOCK protein and mRNA were indeed regulated by ERα (Fig. 2B). Differences in response to E2 between MDA-MB-231 cells (ERβ-positive/ERα-negative) and T47D cells (ERα-positive/ERβ-negative) suggested that ERβ may be less important in the modulation of CLOCK in response to E2, compared to ERα. This was demonstrated by the lack of change in CLOCK protein level in T47D cells overexpressing ERβ (Fig. 2D). These results were consistent with the reporter gene experiments. ICI competes with E2 for binding to ERα and this leads to promotion of ERα degradation, disruption of its localization to the nucleus and subsequent dimerization [51], [58], [59]. In our studies, E2 decreased the expression level of total ERα, but stimulated the expression of CLOCK (Fig. 2C). This result seemed paradoxical considering the relationship between ERα and CLOCK. In fact, ERα tends to accumulate in the nucleus upon E2 stimulation, and may enhance its own transcriptional activity. Subsequently, ERα is ubiquitinated and degraded through the 26S proteasome pathway [60], [61]. Thus a possible mechanism could be that in ERα-positive breast cancer cells, E2 decreases the expression level of ERα, but at the same time, increases its nuclear translocation, resulting in an increased level of ERα in the nucleus and hence enhancement in ERα activity [51].

E2-ERα signaling mediates the transcription of target genes through classical and non-classical pathways. In the classical pathway, ERα binds E2, and becomes dimerized. The dimeric ERα then interacts with the conserved, imperfect or truncated EREs in the promoter or regulatory regions of the target genes to activate or repress their transcription. In the non-classical pathway, ERα modulates gene transcription through interacting with other transcription factors, such as AP-1, NF-κB or Sp1 [52], [53]. Two putative ERα-binding sites were identified in the promoter of CLOCK. The sequence (TGACG) of the site located downstream the TSS site (+2 to +6) was the same as the ERE located in the Metastasis Associated protein 3 (*MTA3*) promoter[62], while the sequence (AGGCCTTGTGACCC) of the other site (+753 to +764) overlapped with the site of RevRE [30]. The overlapping sequence is GTGACCC. The activation of *CLOCK* transcription by ERα was inhibited by the coexpression of REV-ERBα (Fig. 4C). This may be due to the consequence of the interplay between REV-ERBα and ERα, both of which competed for the cis-acting elements in *CLOCK*. The binding of ERα to *CLOCK* promoter was confirmed *in vivo* by ChIP analysis (Fig. 5C). Whether ERα would directly bind to the *CLOCK* promoter *in vitro* will be a subject of further study. Importantly, knocking down the expression of CLOCK attenuated the proliferation of MCF-7 cells, leading to colony formation and soft-agar colony growth (Figs. 6A, C&D). In addition, Brooke H. Miller *et al* reported that *Clock* mutation significantly inhibits the growth and proliferation of fibroblast cells derived from mouse embryos [14]. These results seem to provide evidence for a role of CLOCK in cellular proliferation.

In ERα-positive human telomerase-immortalized breast epithelial cell line, the transcriptions of key clock genes, such as *PER1*, *PER2*, *PER3*, *BMAL1*, *CRY1*, *CRY2* and *Rev-Erbα*, and *ESR1* (*ERα*) were found to display circadian oscillation after entrainment, which was applied using a serum shock method [16], [63]. In contrast, ERα-positive and ERα-negative breast cancer cells show a disrupted inner clock following entrainment [16]. Moreover, *ESR1* mRNA level in ERα-positive breast cancer cells, such as MCF-7 and T47D does not show circadian oscillation [16]. It is attractive to elucidate whether the loss of circadian oscillation of ERα may actually contributes to the abnormal expression of CLOCK and cell proliferation in breast cancer cells. Since CLOCK is a core transcription factor in mammalian circadian clock, it is reasonable to speculate that abnormal activation of E2-ERα signaling could induce the overexpression of CLOCK, and disrupt the circadian clock in breast cancer cells. It is worthwhile to note that the present work examined the transcriptional mechanism of *CLOCK* in ERα-positive breast cancer cells, and tried to determine if a correlation between ERα and CLOCK exists in these cells. Although the data appeared to indicate that ERα played a role in upregulating the expression of CLOCK in ERα-positive breast cancer cells, and that such regulation could be stimulated by E2, whether this mechanism is also important in normal breast cells needs to be addressed by further study.

**Figure 7:**
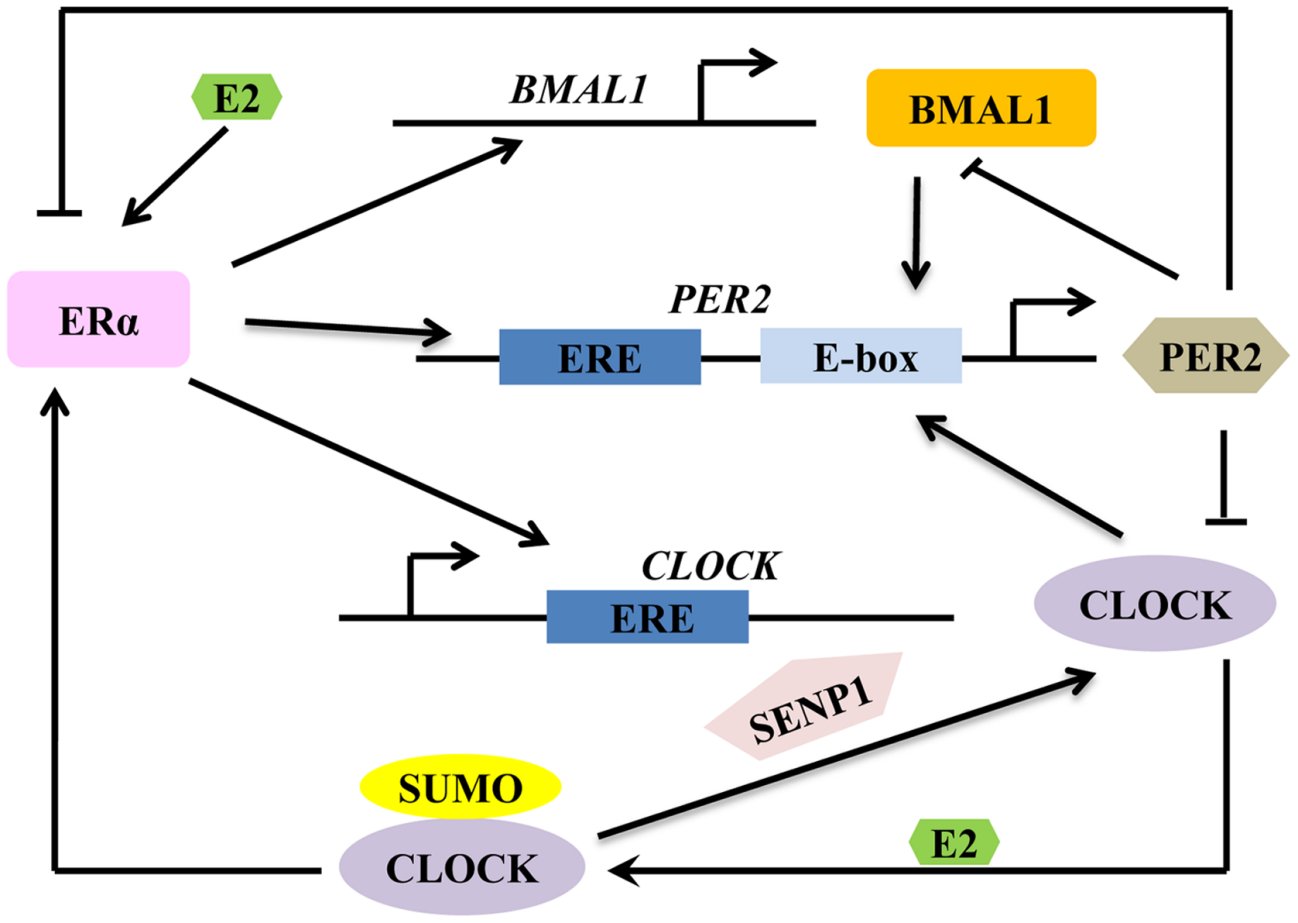
Proposed model showing the crosstalk between E2-ERα signaling and circadian rhythm.

A possible model depicting how E2-ERα signaling is coupled to the machinery of circadian clock is shown in Figure 7. In this model, *CLOCK*, *BMAL1* and *PER2* transcription can be modulated by E2-ERα signaling. E2 enhances the sumoylation of CLOCK and the interaction of CLOCK with ERα. Sumoylated CLOCK may also function to increase the transcriptional activity of ERα. Meanwhile SENP1, which has been identified as a protease that desumoylates CLOCK may play a role in regulating the status of CLOCK sumoylation [27]. E2 also stimulates the transcription of *PER2,* leading to the accumulation of its transcript, and hence increasing the level of PER2 protein in the cytoplasm. PER2 will then be transported into the nucleus where it may inhibit the transcriptional activity of ERα [25]. Overexpression of PER2 has been shown to inhibit cell growth and the rise of clonogenic cells in breast cancer cells [25]. Moreover PER2 also inhibits the transactivation of the circadian proteins, CLOCK and BMAL1. As the whole pathway is a network, the transcription of *BMAL1* is also regulated by E2 and ERα [63]. These previous studies seem to suggest that there is a closely relationship between circadian rhythm and E2-ERα signal pathway. The transcriptional regulation CLOCK mediated by E2-ERα signaling demonstrated in the current study would provide a positive contribution to the further understanding of the molecular mechanism by which E2 alters the circadian rhythm in behavior, physiology, and reproductive functions in mammals. At the same time, the crosstalk between E2-ERα signaling and CLOCK would add to the complexity of the mammalian circadian clock feedback loop.

In conclusion, our results indicated that *CLOCK* is a downstream transcriptional target of ERα, and this provided potential insights into the connections between E2-ERα signaling and circadian rhythm, and showed that *CLOCK* may be an integral part of the series of genes that constitute the responsiveness of cells to the presence of estrogen, functioning as part of the network of transcriptional events governed by ERα. This will serve as a step forward in unraveling the complex mechanisms involved in the development of breast cancer involving a clock gene.
